# Dose calibration of EPIDs for segmented IMRT dosimetry

**DOI:** 10.1120/jacmp.v15i6.4895

**Published:** 2014-11-08

**Authors:** Shrikant Deshpande, Aitang Xing, Lois Holloway, Peter Metcalfe, Philip Vial

**Affiliations:** ^1^ Department of Medical Physics Liverpool and Macarthur Cancer Therapy Centre Sydney Australia; ^2^ Centre for Medical Radiation Physics University of Wollongong Wollongong Australia; ^3^ Ingham Institute for Applied Medical Research Sydney NSW Australia; ^4^ Institute of Medical Physics School of Physics, University of Sydney Sydney Australia; ^5^ South Western Sydney Clinical School University of New South Wales Sydney NSW Australia

**Keywords:** electronic portal imager, flat‐panel imager, amorphous silicon EPID, portal dosimetry, IMRT

## Abstract

The purpose of this study was to investigate the dose response of amorphous silicon (a‐Si) electronic portal imaging devices (EPIDs) under different acquisition settings for both open jaw defined fields and segmented intensity‐modulated radiation therapy (IMRT) fields. Four different EPIDs were used. Two Siemens and one Elekta plus a standalone Perkin Elmer research EPID. Each was operated with different acquisition systems and settings. Dose response linearity was measured for open static jaw defined fields and ‘simple’ segmented IMRT fields for a range of equipment and system settings. Six ‘simple’ segmented IMRT fields were used. The segments of each IMRT field were fixed at $10\times 10\;{\text{cm}}^{2}$ field size with equal MU per segment, each field having a total of 20 MU. Simultaneous measurements with an ionization chamber array (ICA) and EPID were performed to separate beam and detector response characteristics. Three different pixel calibration methods were demonstrated and compared for an example ‘clinical IMRT field’. The dose response with the Elekta EPID for ‘simple’ segmented IMRT fields versus static fields agreed to within 2.5% for monitor unit (MU)≥2. The dose response for the Siemens systems was difficult to interpret due to the poor reproducibility for segmented delivery, at MU≤5, which was not observed with the standalone research EPID nor ICA on the same machine. The dose response measured under different acquisition settings and different linac/EPID combinations matched closely (≤1%), except for the Siemens EPID. Clinical IMRT EPID dosimetry implemented with the different pixel‐to‐dose calibration methods indicated that calibration at 20 MU provides equivalent results to implementing a ghosting correction model. The nonlinear dose response was consistent across both clinical EPIDs and the standalone research EPID, with the exception of the poor reproducibility seen with Siemens EPID images of IMRT fields. The nonlinear dose response was relatively insensitive to acquisition settings and appears to be primarily due to gain ghosting effects. No additional ghosting correction factor is necessary when the pixel‐to‐dose calibration factor at small MU calibration method is used.

PACS numbers: 87.53.Bn, 87.55.Qr, 87.56.Fc, 87.57.uq

## I. INTRODUCTION

All medical linear accelerator (linac) vendors currently provide amorphous silicon (a‐Si) electronic portal imaging devices (EPIDs) as a standard option. EPIDs are used routinely in radiotherapy for image verification of patient position. However, it has been demonstrated previously that a‐Si EPIDs also have great potential for dosimetry applications.^(1)^

Despite many studies demonstrating the potential of a‐Si EPIDs for dosimetry, there remain some technical challenges to be overcome in order to realize their full potential in routine clinical practice. There are issues related to the non‐water equivalence of the EPID^2^, ^3^, ^4^, ^5^ and the detector's image acquisition process.^6^, ^7^, ^8^ Previous studies of the dose response characteristics of a‐Si EPIDs have reported an underresponse at small monitor unit (MU) exposures relative to longer exposures.^9^, ^10^, ^11^, ^12^, ^13^ The resulting nonlinear EPID dose response, referred to here as gain ghosting, has been attributed to trapped charge effects.^9^, ^13^, ^14^, ^15^ Gain ghosting is associated with variations in the quantity of trapped charge with exposure to radiation. The electric field characteristics change as the level of trapped charge increases, resulting in a change in pixel sensitivity with exposure to radiation. Image lag, defined as residual signal registered with a time delay from the original radiation induced electron‐hole pair, is also attributed to trapped charge.^(14)^

Image lag measured from the relative residual signal in image frames acquired immediately after an exposure has been reported as 2%–10%, depending upon incident exposure and EPID model.^9^, ^10^, ^11^, ^13^ McDermott et al.^(9)^ measured both image lag versus time elapsed (postirradiation) and linearity of dose response (gain ghosting) for an Elekta iViewGT a‐Si EPID. They proposed a correction for ‘combined ghosting effects’ to account for both image lag and gain ghosting using a triple exponential fitted as a function of time based on measurements with open beams. The same group quantified the nonlinear response of six EPIDs from three different vendors: Elekta iView (Elekta Oncology Systems, Crawley, UK), Varian aS500 (Varian Medical Systems, Palo Alto, CA), and Siemens OptiVue (Siemens Medical Solutions, Concord, CA).^(10)^ The underresponse was in the order of 4%–6% at 5 MUs relative to 1000 MU for Siemens and Elekta EPIDs. Nijsten et al.^(11)^ also reported an underresponse of up to 6% at 5 MU exposures relative to 1000 MU for a Siemens EPID, and implemented the ghosting correction factor into their EPID dosimetry calibration algorithm as proposed by McDermott et al.^(9)^ Similar nonlinear characteristics were measured on an Elekta EPID by Winkler et al.^(13)^ They proposed that the EPID dose response be a logarithmic function of dose, rather than time as proposed by McDermott et al.^(9)^ This research group accounted for an additional dose rate response effect during linac beam startup and demonstrated that image lag increases with the ratio of MUs between the first and second exposure, and with reduced time interval between two subsequent exposures for intensity‐modulated radiotherapy (IMRT) fields. Recently, Warkentin et al.^(16)^ proposed a pixel‐by‐pixel correction model that incorporates both image lag and nonlinearity correction for dynamic delivery with a Varian aS500 system. In the same study, they highlighted the importance of these corrections to reduce ambiguities and uncertainties in EPID‐based dose verification. Van Esch et al.^(17)^ reported a forgoing irradiation of 500 MU resulted in image lag of only 1% in the following image acquired after approximately 10 s for Varian aS500 EPIDs. They also attributed the underresponse of up to 6% at 2 MU mainly to rounding error of signal count from the acquisition software. Another factor in EPID dose response, which has not been addressed in most studies, is the importance of different image acquisition software controls and frame readout schemes. Chang and Ling^(6)^ identified potential errors in the Varian synchronous, frame‐averaging acquisition mode due to missing data between the start of irradiation and imaging, and from the last (incomplete) frame. Kavuma et al.^(7)^ also observed significant artifact in in‐plane profiles at low MU exposures on Varian EPIDs with IAS2/IDU‐II acquisition software, and suggested the IAS2/IDU‐II acquisition system would not be suitable for step and shoot IMRT verification with low MU segments. Both of these issues were resolved in following vendor upgrades. Budgell et al.^(18)^ investigated the intersegment EPID response reproducibility at low dose measured over a series of 20 successive segments delivered with 1 and 2 MUs. The measured inter segment variability was within 1% and consistent with ion chamber data. They also reported the acceptable reproducibility of off‐axis profiles measured for 20 successive segments with 4 MU. A comprehensive investigation about the influence of the readout scheme on the dose response for all three linac vendors at small MU was carried out by Podesta et al.^(8)^ This research group modeled the discrepancies in dose response at low MU of up to 37% using only the incomplete integration of EPID frames acquired during irradiation. They reported no underresponse for Elekta and Varian TrueBeam systems (postsoftware upgrade), but reported large underresponse for Siemens, Varian TrueBeam (presoftware upgrade) and Varian Clinac systems. While difficult to compare directly, these results do not appear consistent with previous EPID dose response studies.

The above review summarizes some of the key studies of EPID dose response, highlighting the inconsistent interpretation of EPID dose response characteristics. Despite the apparent successful and increasingly widespread clinical implementation of EPID dosimetry, fundamental dose response issues remain unresolved. These issues came to light during the development of an EPID dosimetry program in our department and were the motivation for this work. The specific issues we aim to address in this work include: i) the inconsistencies in the literature about the underlying causes of nonlinear dose response of EPIDs; ii) the EPIDs nonlinear behavior is widely reported based on static open beam exposures, with little or no consideration for how accurately this behavior translates to segmented IMRT; iii) the management of these effects on a multivendor EPID dosimetry program, with particular regard to the relative importance of the EPID detector design, the linac, and the image acquisition and processing methods implemented across different systems; and iv) unexpected EPID dose response behavior observed on Siemens EPIDs. This work will contribute to a more consistent understanding and implementation of pixel‐to‐dose calibration methods for EPID‐based IMRT dosimetry.

## II. MATERIALS AND METHODS

### A. Equipment

Different combinations of linacs, EPIDs, and acquisition software were investigated in an attempt to isolate the source and relative contributions to EPID dose response behavior. Each experimental setup is described in Table 1. The bottom row provides a code used to refer to each experimental setup throughout this paper. In each case the standard gain (flood field) and offset (dark field) corrections were applied to EPID measurements. All IMRT fields were delivered using the segmented (step and shoot) technique with gantry angle fixed at 0° with 6 MV photons only.

**Table 1:** The combinations of equipment and settings for each experiment.

	*Linac 1*	*Linac 2*		*Linac 3*		
Linac Model	Siemens Oncor	Siemens Oncor		Elekta Synergy		
Dose Rate (MU/min)	300	300		500		
EPID Model	Optivue 1000	Optivue 1000ST	Research	iViewGT		Research
	P.E. XRD 1640 AL7	P.E. XRD 1640 AG9	P.E. XRD 1640 AN CS	P.E. XRD 1640 AL5 P		P.E. XRD 1640 AN CS
Source to Imager distance (cm)	115	115	115	160		160
Acquisition Software	Coherence Therapist	Coherence Therapist	P.E.XIS	Elekta iViewGT	P.E.XIS	P.E.XIS
	v.2.1.24	v.2.1.24	v.3.3.1.1	v.3.4b 162‐SP2	v.3.2.0.7	v.3.3.1.1
EPID integration time (ms)	285	143	133	433	433	433
ICA integration Time(ms)	280	140	130	430	430	430
Experiment setup	1	2a	2b	3a	3b	3c

P.E. = PerkinElmer, Santa Clara, CA; ICA ionization chamber array (IBA Dosimetry Asia Pacific, Beijing, China).

#### A.1 Siemens EPID system

Details of EPID construction, acquisition software, and image processing implemented on the Siemens equipment (Siemens Medical Solutions, Concord, CA) can be found in previous study.^(11)^ The EPID images for step and shoot IMRT delivery were acquired with the multi‐frame acquisition mode (experimental setup 1 and 2a from Table 1). This mode of acquisition saves a frame average image for each IMRT segment. According to the Siemens documentation,^*^ the number of frames ($N_{\text{frames}}$) acquired per segment or beam is determined by the following relation:

(1) $$N_{f\;\text{rames}}=\frac{\text{ExpectedDose}(\text{MU})\times 60(\text{sec}/\text{min})}{\text{DoseRate}(\text{MU}/\text{min})\times \text{IntegrationTime}(\text{sec})}$$

The integrated pixel value (IPV) for IMRT fields with N segments is given by Eq. (2):

(2) $$\text{Integrated}\;\text{Pixel}\;\text{Value}=\text{Integrated}\;\text{Pixel}\;\text{Value}=\sum_{i=1}^{n}\left(R_{i}\times N_{i}\right)$$

where, $R_{t}$ denotes the EPID frame average pixel value for the ith segment (i.e., EPID frame average response per segment), and $N_{i}$ denotes the number of frames ($N_{\text{frames}}$) for the ith segment. $N_{\text{frames}}$ is reported in the DICOM file header. All images were exported in DICOM format and analyzed with in‐house code using MATLAB (MathWorks Inc version 7.12.0.635(R2011a); MathWorks, Natick, MA). The ‘Port during’ imaging option used for IMRT operates in ‘free running mode’. Based on personal communications^†^ with other research groups and the vendor, the image acquisition is thought to be triggered by a beam‐on and beam‐off signal from secondary Siemens software. There is no beam pulse synchronization in ‘free running mode’.

#### A.2 Elekta EPID system

Details of EPID construction, acquisition software, and image processing implemented on the Elekta equipment (Elekta Oncology Systems, Crawley, UK) can be found in previous studies.^9^, ^13^ Measurements with the Elekta EPID (experimental setup 3a and 3b from Table 1) were conducted using two image acquisition software systems available on the iViewGT workstation: i) iViewGT Elekta software in standard clinical mode (version 3.4b 162‐SP2), and ii) XIS PerkinElmer software (version 3.2.0.7). IMRT images acquired with the iViewGT software use the ‘Single’ exposure option. This mode of acquisition saves a frame average image for each segment. Individual frame average image for each segment was exported. The integrated pixel value (IPV) for each segment or field is obtained using Eq. (3):

(3) $$\text{Integrated pixel value}=\frac{65535-\text{RawPixelvalue}}{\text{PSF}}$$

where the 65535 is the 16‐bit offset and *PSF* is the pixel scaling factor. The PSF for each segment of an IMRT field is reported by the iViewGT software. The integrated EPID image was obtained by manually adding the IPV of each segment determined using Eq. (3). The PSF includes scaling factors for the number of frames acquired and a configurable renormalization used to ensure gray‐scale values are optimal for visualization of clinical images. When this renormalization is set as 0 in the iViewGT initialization file (sri.ini), the PSF is numerically equal to the inverse of the number of frames acquired during the image, analogous to $1/N_{\text{frames}}$ from Eq. (1). The number of frames acquired PostBeamOff is also configurable in the initialization file. To investigate the impact of this setting on pixel‐to‐dose calibration, the EPID images were collected with zero, three, and ten PostBeamOff frames with renormalization settings of 40,000 (the default clinical setting) and zero. The images and associated log files (containing the image header information) were exported using the standard export option from the iViewGT workstation for further analysis. The image data controller uses the gun pulses from the linac to synchronize the reading of the data from the panel, so that image data is read when radiation pulse is not present. As soon as the complete frame is read from the panel, a frame synchronization pulse is sent to the data controller.^‡^

**Figure 1:**
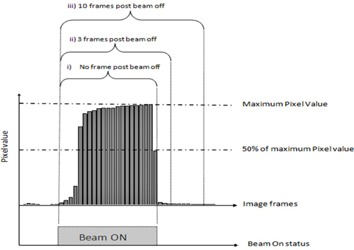
Schematic illustration of three different frame integration methods: i) No frame PostBeamoff; ii) 3 frames PostBeamoff; and iii) 10 frames PostBeamoff, where 0, 3, and 10 frames after the beam has stopped are included in the integrated images, respectively. The beam‐off trigger was estimated as the frame where the signal had dropped to approximately 50% or less of the beam‐on value.

The measurements with the XIS application (experimental setup 3b from Table 1) were conducted in continuous ‘free running mode’ with no external trigger mechanism. The acquisition was manually started immediately prior to beam‐on and at an arbitrary time after beam‐off (at least 10 frames after beam‐off). The XIS software stores the individual frames. The integrated image was obtained by summing each frame in MATLAB. The frame signal amplitude was used to indicate the start and stop of each beam and hence control the number of PostBeamOff frames used in the analysis (Fig. 1). The two different acquisition systems (iViewGT and XIS) on the same linac enabled us to investigate the impact of software based acquisition settings on the reported dose response of the EPID.

#### A.3 Standalone research EPID system

A standalone PerkinElmer detector was also used in this study. This EPID was similar to the Elekta and Siemens EPIDs, with the advantage of being mobile so it could be used across the different linacs. The research EPID measurements (experimental setup 2b and 3c from Table 1) were conducted on both Siemens and Elekta linacs in continuous free‐running mode using XIS acquisition software. The acquisition settings were set to match the clinical acquisition settings as described Table 1. The integrated image was obtained by summing each frame in MATLAB, as described above for the Elekta images acquired with XIS software. The beam‐off trigger was estimated as the frame where the signal had dropped to approximately 50% or less of the beam‐on value. Figure 1 describes the image acquisition and triggering process. Using one EPID across different linacs provided more information on the impact of the linac on the reported dose response of the EPID. For segmented delivery, the ‘10 frames PostBeamOff’ integrated image were obtained by summing the maximum number of PostBeamOff frames available between two consecutive IMRT segments, not strictly 10 frames.

**Figure 2:**
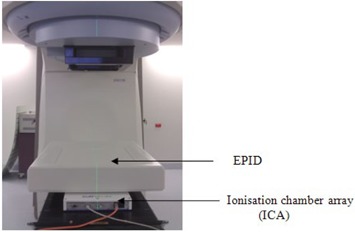
Experimental setup for simultaneous EPID and ionization chamber array measurements.

In order to isolate EPID dose response behavior from beam characteristics, such as dose per MU linearity, each experiment was conducted with the ion chamber array (ICA) (IBA Dosimetry Asia Pacific, Beijing, China) positioned beneath the EPID to acquire simultaneous reference measurements. This ICA has been previously shown to display linear dose response behavior in both ‘movie’ mode and integrating mode.^19^, ^20^ The ICA was operated in ‘movie’ mode at approximately the same integration time as the EPID in each experiment. The ICA software restricts integration times to multiples of ten; therefore, the ICA integration time was set to the EPID integration time rounded to the nearest 10 milliseconds (see in Table 1). The setup for the simultaneous EPID and ICA measurements is shown in Fig. 2. Previous work verified this setup had no effect on the EPID dose response characteristics under investigation.^(21)^

All the measurements discussed below in Material & Methods sections B and C were conducted with all three equipment combinations summarized in Table 1. All the measurements in sections B, C, and D were performed with the simultaneous EPID and ICA setup as shown in Fig. 2. All measurements were conducted with 6 MV photon beam and 3 min intervals between each measurement, unless stated otherwise, to minimize image lag.

### B. Dose linearity for segmented fields

The linearity of EPID response with dose was measured with static open fields for varying MU exposures (1–100 MUs). The open beam experiment provided a baseline for comparison with EPID dose response linearity with IMRT delivery. Six IMRT fields were created specifically for this experiment. The segments of each IMRT field were fixed at $10\times 10\;{\text{cm}}^{2}$ field size, each field having a total of 20 MU. Field 1 consisted of 20 segments at 1 MU per segment, field 2 consisted of 10 segments at 2 MU per segment, and fields 3, 4, 5, and 6 consisted of 5, 4, 2, and 1 segments of 4, 5, 10, and 20 MU per segment, respectively. The 20 MU per segment ‘simple’ IMRT field was analogous to a standard static open beam delivery of 20 MU. The total integrated EPID response for each IMRT field was obtained from the sum of the average of the central 20×20 pixels per frame. The integrated pixel value per segment and in total for each IMRT field was compared with integrated pixel value from a static open beam with the corresponding MUs. For the remainder of this study we refer to the IMRT fields used in this experiment as ‘simple’ IMRT fields.

The average integrated measured values for each experiment was analyzed in the following manner to derive linearity plots: i) ICA measurements (readout value per MU) for each segment and/or field were normalized to the ICA measured value for 100 MU static open beam exposure; ii) the EPID measurements (pixel value per MU) for each segment and/or field were normalized to the EPID‐measured value for 100 MU static open beam exposure; iii) the EPID data from step ii) was again normalized against the corresponding measured ICA data from step i). Step iii) removes any nonlinearity in dose per MU delivery at small MUs from the EPID analysis. All ICA and EPID measurements were conducted simultaneously, as described earlier, to further minimize the impact of any inter‐beam linac output fluctuations on the EPID experiments. The relative EPID response as a function of dose for single static open fields and segmented IMRT fields was compared to assess whether the underresponse seen in static fields persists with segmented IMRT delivery.

### C. Intersegment reproducibility

To evaluate intersegment variations in clinical EPID response during segmented delivery, the ‘simple IMRT fields’ described above were delivered five times consecutively (experimental setup 2a and 3a from Table 1). The reproducibility of EPID response at off‐axis positions was also investigated from profiles along the central pixel row and column for each segment. The ICA provided a reference of delivered dose profiles.

### D. Clinical IMRT fields

Four sets of ‘clinical IMRT fields’ with constant MU per segment of 2, 4, 5, and 10 MUs were created by editing the radiotherapy treatment plan DICOM file. The ‘clinical IMRT fields’ used here were created from a single highly modulated field from a nasopharynx IMRT plan generated by the CMS XiO treatment planning system (CMS Inc., St. Louis, MO). The ‘clinical IMRT fields’ consist of 20 IMRT segments. Ten repeat measurements for ‘clinical IMRT fields’ were performed simultaneously on both Siemens and Elekta clinical EPID along with ICA (Fig. 2). The percentage deviation in the integrated detector response at each pixel was determined from 10 repeat measurements (experimental setup 1,2a, and 3a from Table 1).

### E. EPID pixel‐dose calibration

The integrated EPID dose for the ‘clinical IMRT fields’, described in D above, was determined using three different methods as described by Eqs. (4), (5), (6) below. The results were compared to assess the impact of calibration methodology.

#### E.1 Calibration method 1 (ghosting correction method)

(4) $$\text{Integrated EPID Dose}=\sum_{i=1}^{n}\left[\frac{{\left(\text{IPV}\right)}_{n}}{G{\left(t_{\text{rad}}\right)}_{n}}\right]*(\text{CF}(100\text{MU}))$$

#### E.2 Calibration method 2 (no ghosting correction)

(5) $$\text{Integrated EPID Dose}=\left[\sum_{i=1}^{n}{\left(\text{IPV}\right)}_{n}\right]\times (\text{CF}(100\text{MU}))$$

#### E.3 Calibration method 3 (small MU calibration method)

(6) $$\text{Integrated EPID Dose}=\left[\sum_{i=1}^{n}{\left(\text{IPV}\right)}_{n}\right]\times (\text{CF}(20\text{MU}))$$

where $({\text{IPV})}_{n}$ is the integrated pixel value for the nth segment, CF(100MU) and CF(20MU) is a pixel‐to‐dose calibration factor determined at 100 MU and 20 MU, respectively, $t_{\text{rad}}$ is radiation beam‐on time, and $G(t_{\text{rad}})$ is a ghosting correction factor determined from the function of EPID dose response linearity measured with open static fields for linac 1 and linac 3 (experimental setup 1 and 3a from Table 1). The ghosting correction factor was determined from a third‐order polynomial curve fitted to the EPID dose response as a function of beam‐on time, similar to previous studies.^9^, ^11^ There was no image lag correction applied. The value of $t_{\text{rad}}$ is determined from the product of the number of frames and frame acquisition rate for the Siemens linac and from an inverse of the product of PSF and frame acquisition rate for the Elekta linac (experimental setup 3a from Table 1). The $t_{\text{rad}}$ value for the Elekta EPID system was based on EPID dose response data acquired with the renormalization value set to 0. There is no correction for any specific ghosting or nonlinearity in calibration methods 2 and 3. Method 3 uses a calibration factor determined at a MU level more closely matched to IMRT segment MU, with the aim of reducing the impact of nonlinear dose response (gain ghosting) on clinical dosimetry. A gamma evaluation, with 2% and 3% dose difference (global maximum) and 2 and 3 mm distance‐to‐agreement criterion with 10% dose threshold, is performed to quantify the difference between the integrated EPID dose map calculated from calibration methods 1, 2, and 3 using the same EPID data. Field size correction factors and other detector scatter corrections were ignored for the EPID dose computation, since they remain constant irrespective of calibration methods being investigated here.

## III. RESULTS

### A. Dose linearity for segmented fields

**Figure 3:**
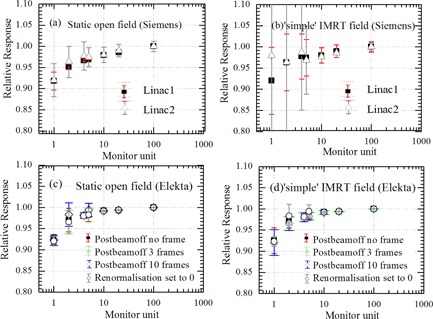
Relative EPID response versus MU for open static and ‘simple’ IMRT fields with fixed field size $10\times 10\;{\text{cm}}^{2}$ for Siemens and Elekta clinical EPID systems (experimental setup 1, 2a, and 3a from Table 1). Data points are the ratio of EPID and ICA response for both static and ‘simple’ IMRT fields. Error bars show the standard deviation from five repeat measurements. All the data points are normalized to nonsegmented single exposure of 100 MU. The renormalization (acquisition setting in sri.ini file) for each of the three different Postbeamoff frame Elekta datasets was set to 40,000.

The EPID dose response as a function of MU for static and ‘simple’ IMRT fields for all EPIDs are shown in Figs. 3(a) and 3(b) for the Siemens linac and Figs. 3(c) and 3(d) for the Elekta linac (experimental setup 1, 2a, and 3a from Table 1). In the case of the Elekta EPID, dose response for ‘simple’ IMRT fields versus static fields agreed to within 2.5% for MU≥2 for 0, 3, and 10 PostBeamOff frames with renormalization setting 40,000 plus renormalization value set to zero. However, for Siemens EPID, the agreement in dose response for ‘simple’ IMRT fields versus static fields was difficult to interpret due to the poor reproducibility for segmented delivery, particularly at MU≤5. Both Siemens (for static open jaw defined fields) underresponded by 9%, while Elekta clinical EPIDs underresponded by 8% relative to ICA for 1 MU per segment (or field). At 2 MU and above, the agreement between Elekta clinical EPID and ICA was within 2.5%. No significant difference was observed in dose response for the Elekta clinical EPID when measured under different acquisition setting, as described in the Methods & Materials section A, and agreed within 1%. The renormalization for each of the three different PostBeamOff frame Elekta datasets was set to 40,000 (Figs. 3(c) and (d)). At 2 MU and above, the agreement between Siemens clinical EPID and ICA was within 3.5%. The reproducibility at 1 MU for clinical Siemens EPIDs was poor (7.8% and 12.7% SD for linac 1 and linac 2, respectively; see Fig. 3(c)), while for clinical Elekta reproducibility was clinically acceptable (<3.0% SD for all acquisition setting). The reproducibility of the ICA data remained within 0.3% in all cases.

**Figure 4:**
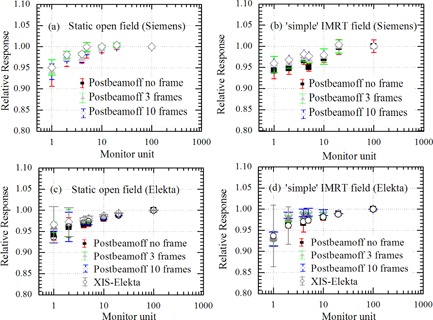
Relative EPID response versus MU for open static and ‘simple’ IMRT fields with fixed field size $10\times 10\;{\text{cm}}^{2}$ for research EPID measured on Siemens and Elekta linacs (experimental setup 2b and 3c from Table 1). Relative EPID response for clinical Elekta EPID using XIS software (experimental set up 3b from Table 1). Data points are the ratio of EPID and ICA response for both static and ‘simple’ IMRT fields. Error bars show the standard deviation from five repeat measurements. All the data points are normalized to nonsegmented single exposure of 100 MU.

**Figure 5:**
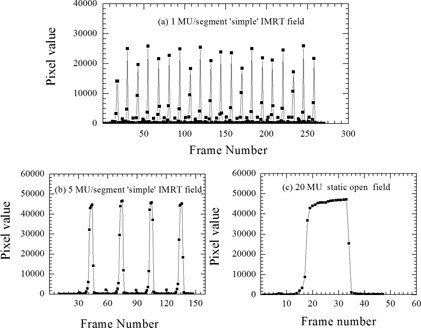
The EPID response at CAX per frame for ‘simple’ IMRT fields for (a) 1 MU and (b) 5 MU per segment, respectively; (c) static open field (20 MU).

The EPID dose response as a function of MU for static and ‘simple’ IMRT fields for the research EPID are shown in Figs. 4(a) and 4(b) for the Siemens linac and Figs. 4(c) and 4(d) for the Elekta linac (experimental setup 2b and 3c from Table 1). In both cases, the EPID dose response for ‘simple’ IMRT fields versus static fields agreed to within 1.3% for MU≥2. The research EPID underresponded by 6% and 5% relative to ICA for 1 MU per segment for Siemens and Elekta linacs, respectively. At 4 MU and above, the agreement between research EPID and ICA response was within 3% for both linacs. The dose response measured with the clinical Siemens EPID agreed closest with the dose response measured with the research EPID with no PostBeamOff frame setting for static open fields. The research EPID did not show the poor reproducibility seen at small MU with the Siemens EPID (Fig. 3), confirming this was not related to the beam. The dose response measured with clinical Elekta EPID agreed with dose response measured with the research EPID to within 1% for both static and ‘simple’ IMRT fields. Figure 5 depicts the EPID pixel signal collected frame by frame at the central axis. Table 2 compares the integrated EPID response for single 20 MU exposure with total integrated EPID response for ‘simple’ IMRT fields for 1 MU, 2 MU, 4 MU, 5 MU, and 10 MU per segments on linac 2 and linac 3. Table 2 shows the underresponse persists for segmented delivery with 1MU(≤4%–6%) and 2MU(≤2%–3%) per segment, similar to static open. The underresponse was seen for all three PostBeamOff frame settings.

**Table 2:** The integrated research EPID response at CAX for both static and ‘simple’ IMRT fields with total of 20 MU exposure. The captured frames were summed as no PostBeamOff, three PostBeamOff, and ten post‐beam‐off frames for the same EPID image to demonstrate effect from different acquisition protocols. The percentage values in brackets indicate the relative difference in the integrated pixel values for each simple IMRT field compared the static field exposure of the same total dose.

*Linac*	*PostBeamOff Frames Number*				*Mean IPV*		
		Static field		‘simple’	IMRT fields		
		20 MU Single exposure	20×1MU segments	10×2MU segments	5×4MU segments	4×5MU segments	2×10MU segments
	0	765264	718307.1(−6.14%)	736394.2(−3.77%)	745395.4(−2.60%)	751777.7(−1.76%)	760431.3(−0.63%)
Elekta	3	767489.4	723304.2(−5.76%)	746969.1(−2.66%)	757629.8(−1.28%)	757975.9(−1.24%)	764746.9(−0.36%)
	10	769631.1	726087.9(−5.66%)	753367.0(−2.11%)	764005.2(−0.73%)	764841.1(−0.62%)	769548.7(−0.01%)
	0	867426.2	821950.5(−5.24%)	847584.3(−2.29%)	860657.2(−0.78%)	861173.2(−0.72%)	861875.4(−0.64%)
Siemens	3	869129.9	833360.2(−4.12%)	858665.3(−1.20%)	864984(−0.48%)	865182.5(−0.45%)	865338.6(−0.44%)
	10	869129.9	839021.7(−4.05%)	868133.3(−0.72%)	870569.6(−0.44%)	870815.8(−0.41%)	870975.5(−0.39%)

### B. Intersegment reproducibility

#### B.1 Central axis

**Figure 6:**
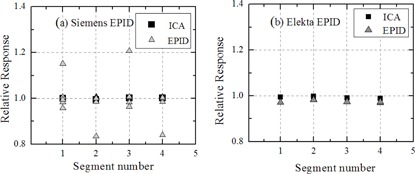
Relative response per segment for five repeats of the ‘simple’ IMRT field for both clinical EPID (Siemens and Elekta) measured with 5 MU/segment (experimental setup 2a and 3a from Table 1). All the data points are normalized to nonsegmented single exposure 20 MU.

Figure 6 depicts the integrated dose response per segment at the central axis measured with both the clinical EPIDs and ICA for the ‘simple’ IMRT field with 5 MU per segment (experimental setup 1 and 3a from Table 1). The large variations in Siemens EPID response reflect the large errors bars in Fig. 3(b). The variation in Siemens EPID response per segment was up to ±20%. The simultaneously measured ICA did not indicate any variation in beam delivery. The Elekta EPID response per segment was within 1%. Further investigation for the Siemens EPID images found inconsistencies in the value of $N_{\text{frames}}$. The frequency of this EPID dose response phenomenon varied depending on the MU per segment. Intersegment reproducibility was worst at MU per segment ≤5MU; at 10 MU per segment and above the phenomena was not observed at all and reproducibility was within 1%. The same variation in $N_{\text{frames}}$ was not present for the measurements taken with the same linac, EPID, and software for open static beam (nonsegmented) delivery at any MU settings.

#### B.2 Off‐axis

The off–axis profiles for each IMRT segment (frame average image) along the central row (cross‐plane) and central column (in‐plane) of the Siemens EPID panel and ICA were compared for the ‘simple’ IMRT fields. Some EPID cross‐plane profiles were tapered at the field edge, while the in‐plane profiles did not display this effect. By comparison, the profiles in both planes (experimental setup 1 and 2a from Table 1) measured simultaneously with the ICA did not show any tapering of profile shape or any variation in amplitude acquired within segments. No such artifacts in profiles were observed in clinical Elekta EPIDs (data not shown).

### C. Clinical IMRT field

**Figure 7:**
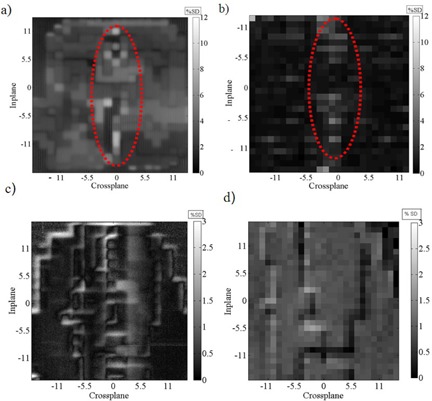
2D maps of the percentage standard deviation at each pixel/detector position within the field from ten repeats for a ‘clinical’ IMRT field with 5 MU per segment (a) clinical Siemens EPID (c) clinical Elekta EPID and (b) and (d) for I'mRT Matrixx detector measured with Siemens and Elekta linac simultaneously.

Figure 7 shows a 2D map representing the percentage standard deviation at each pixel for both clinical EPIDs (Siemens and Elekta) and the ICA for the same ‘clinical IMRT field’ from ten sequential measurements acquired simultaneously on the two detectors (experimental setup 1 and 3a from Table 1). The figures have been scaled and cropped to show the same spatial extent of the IMRT field. The average percentage standard deviation of the entire region of data shown in Figs. 7(a) and (b) was 3.98% (0.90%), 2.94% (0.95%), 2.34% (0.68%), and 1.55% (0.17%) for the EPID (ICA) measurements with 2, 4, 5, and 10 MU per segment cases, respectively, for Siemens EPID. The average percentage standard deviation of the entire region of data shown in Figs. 7(c) and (d) was 1.6% (0.90%), 1.4% (0.85%), 1.3% (0.70%), and 1.05% (0.18%) for the EPID (ICA) measurements with 2, 4, 5, and 10 MU per segment cases, respectively, for Elekta EPID. The maximum percentage standard deviation of the entire region of data 9.2% (3.5%), 8.1% (2.7%), 7.5% (2.2%), and 1.9% (1.4%) for Siemens (Elekta) EPID measurements with 2, 4, 5, and 10 MU per segment cases, respectively. The gray scale image provides a visualization of the poorer reproducibility of the Siemens EPID measurements compared to the ICA.

### D. EPID pixel‐dose calibration

To determine the ghosting correction factor, $G(t_{\text{rad}})$, described in the Material & Method section E and implemented in calibration method 1 (Eq. (4)), a curve (polynomial function) was fitted to both the Siemens and Elekta clinical EPIDs static beam exposure dose response (Figs. 3(a) and 3(c)) as a function of beam‐on time. The polynomial curve fitted to the Elekta EPID dose response as a function of beam‐on time with renormalization setting 0 and 4000 agreed within 1% and, therefore, the ghosting correction $G(t_{\text{rad}})$ for Elekta EPID was used based on EPID dose response data acquired with the renormalization value set to 0. The function fit was accurate within 0.5% to measured data for beam‐on time (down to 2 MU). Table 3 depicts the percentage gamma pass rate for the ‘clinical’ IMRT (experimental setup 1 and 3a from Table 1) when the EPID dose calculated using the method 1 (Eq. (4)) against the EPID dose calculated using method 2 and 3 (Eqs. (5), (6)).

**Table 3:** The percentage gamma pass rate determined using three different calibration methods for both Siemens and Elekta EPIDs for the same integrated image of an IMRT field. The IMRT fields were modified to have a fixed number of MU per segment. The Elekta EPID images were acquired with clinical setting (i.e., renormalization set to 40,000) and for two different PostBeamoff acquisition settings.

		*Percentage Gamma Pass rate*			
*Calibration Methods*	*Clinical IMRT (MU/segment)*	*Siemens*		*Elekta*	
		*2%/2 mm*	*3%/3 mm*	*2%/2 mm*	*3%/3 mm*
Method1 vs. Method2		64.28%	92.69%	99.83%^a^	99.98%^a^
	2MU			99.71%^b^	99.96%^b^
Method1 vs. Method3		92.25%	99.98%	99.73%^a^	99.97%^a^
				99.90%^b^	100.0%^b^
Method1 vs. Method2		85.75%	99.87%	100%^a^	100%^a^
	4MU			99.99%^b^	100%^b^
Method1 vs. Method3		100%	100%	100%^a^	100%^a^
				100%^b^	100%^b^

^a^ Three PostBeamOff frames.

^b^ Zero PostBeamOff frames.

## IV. DISCUSSION

The dose response linearity experiments conducted in this work confirm that the nonlinear dose response measured with static open beams at small MU persists in segmented IMRT delivery based on the MU per segment. This validates the pixel‐to‐dose calibration methods incorporating a ghosting correction on a segment‐by‐segment basis for IMRT dosimetry using corrections determined from open static beam exposures. The existence of gain ghosting was confirmed as being present and consistent across different EPIDs and linear accelerators at small MU exposure. Gain ghosting is associated with variations in the quantity of trapped charge with exposure to radiation. The rate of the accumulation of trapped charge and the rate of signal from the discharge of trapped charge slowly approaches equilibrium with increasing dose. The close agreement between segmental IMRT and static open beam delivery, and the fact that the static open beam fields had substantially longer time periods between subsequent exposures, supports the finding that gain ghosting affects dominate over image lag signal from previous exposures in terms of the EPIDs nonlinear response behavior at small MU. The nonlinearity at small MUs was relatively insensitive to acquisition settings. We have not investigated the impact of pulse repetition frequency (PRF). A method of simultaneous measurements with an ICA and EPID was demonstrated to reliably separate out the EPID dose response from beam delivery characteristics. This methodology demonstrated an irregularity in the Siemens implementation, which resulted in poor measurement reproducibility of IMRT fields at small MU per segment. It was also demonstrated that the ghosting correction is not required if the EPID is calibrated at an appropriately small MU exposure.

The magnitude of underdose response reported in the present work is smaller than some previously reported studies. This may be partly due to the fact we normalized the dose response relative to 100 MU rather than 1000 MU used in other studies.^10^, ^11^ In previous work^(22)^ on Siemens linacs, we normalized to 800 MU and achieved larger underdose response in closer agreement with other studies.^(11)^ At 2 MU and above, the agreement between EPID and ICA response was within 2.5% (for Elekta) and 3.5% (for Siemens). The dose response measured with clinical EPID and the default clinical software setting shown in Fig. 3 agreed with the research EPID dose response shown in Fig. 4. No significant difference (≤1%) was observed in dose response for both the static and ‘simple’ IMRT fields measured with the clinical Elekta EPID under different PostBeamOff frame acquisition settings. This demonstrates that nonlinearity at small MUs was relatively insensitive to acquisition settings and across different EPID detectors. The poor reproducibility for the Siemens EPID in the case of ‘simple’ IMRT fields was not observed either with simultaneous measurement conducted, neither with ICA nor with the standalone research EPID (Fig. 4). There were particularly large variations in the integrated Siemens EPID response between successive segments having 2, 4, and 5 MU per segment, as shown in Fig. 6. Intersegment reproducibility was worst at MU per segment ≤5MU for Siemens EPIDs and occurs in a nondeterministic manner. This was attributed to an inconsistent number of frames per segment being recorded in the image header file. The vendor documentation indicates that the frame number is derived using Eq. (1). This equation appears to be independent of actual delivery parameters that may affect the real number of frames acquired, such as real dose rate variations, and should therefore not vary for a given MU per segment. The variation in integrated EPID response was not seen for static beam (non‐IMRT) exposures on the same linac. We also observed the tapered shape or variation in amplitude acquired within segments for ‘simple’ IMRT fields in case of Siemens EPID only. This variation in amplitude may be due to the synchronization between linac trigger pulse (beam‐on and beam‐off) and detector control board incorporating the real‐time dose‐rate variation, particularly for segment IMRT delivery. The experiment performed on both Siemens EPIDs (experimental setup 1 and 2a from Table 1) shows inconsistent reporting of frame number in the header file, suggesting that it is an issue with Siemens image acquisition systems for segmented delivery. Moreover, Podesta et al.^(8)^ modeled the discrepancies in Siemens clinical EPID dose response up to 37% at 1 MU and 20% at 2 MU. The author validated that these variation are associated with acquisition readout scheme (i.e., missing frames). We have not addressed the additional scatter or spectral corrections that can be applied to account for patient transit or MLC transmission effects.^5^, ^11^ Dynamic MLC and VMAT were not available for this study, and further work is required to determine how these results apply to that setting. The dose linearity response reported in this study is only at 6 MV. The dose linearity at 18 MV in our previous study^(22)^ for Siemens EPID was within 2% compared to 6 MV beam at ≥1MU. Winkler et al.^(13)^ also reported the EPID dose response for 6, 10, and 25 MV photons with an Elekta system and confirmed the dose linearity of the EPID did not depend on energy.

The ghosting correction suggested by McDermott et al.^(9)^ and Nijsten et al.^(11)^ was designed to keep the EPID measured dose accuracy at lower MUs within approximately 1%. The ghosting correction factor was determined as a function fit to a dose‐response curve that ranged from 5 MU to 1000 MU, normalized to 1000 MU. This range of MU does not reflect clinical step and shoot IMRT technique which may be delivered with fewer than 5 MU per segment and is rarely delivered with more than 30 MU per segment for conventional dose fractionation. We selected 20 MU for calibration in our study for two reasons: i) the mode value for maximum MU per segment in most of IMRT fields is nominally 20 MU; and ii) linac output is relatively stable prior to 20 MU being delivered. However based on the dose‐response curve in Figs. 3(a) and 3(c), in principle, calibration value at 4 or 5 MU can be used, provided the linac output is stable. Ghosting and image lag corrections are complex to implement accurately on a pixel‐by‐pixel basis due to the variations in time between segments and variation in dose per segment. For step and shoot delivery, based on our results, we can conclude that adding a ghosting or image lag correction is an unnecessary complication for accurate EPID based IMRT dosimetry.

## V. CONCLUSIONS

The EPID dose response behavior for step and shoot IMRT fields delivered by Siemens and Elekta linacs was investigated. The nonlinear EPID dose response as a function of MU measured for single open beam exposures was found to be consistent with dose response for segmented IMRT delivery. The nonlinear dose response was consistent across both clinical EPIDs and the standalone research EPID, with the exception of the poor reproducibility seen with Siemens EPID images of IMRT fields. The nonlinear dose response was relatively insensitive to acquisition settings and appears to be primarily due to gain ghosting affects in the a‐Si photodiodes. When the pixel‐to‐dose calibration factor was determined at 20 MU, no additional ghosting correction factor is necessary for the accurate determination of dose for clinical IMRT fields.
